# Analysis of protein structure changes and quality regulation of surimi during gelation based on infrared spectroscopy and microscopic imaging

**DOI:** 10.1038/s41598-018-23645-3

**Published:** 2018-04-03

**Authors:** Wei Wei, Wei Hu, Xian-Yi Zhang, Feng-Ping Zhang, Su-Qin Sun, Yuan Liu, Chang-Hua Xu

**Affiliations:** 1 College of Food Science & Technology, Shanghai Ocean University, Shanghai, 201306 P. R. China; 2 Tongwei Co., Ltd., Chengdu, Sichuang, 610041 P. R. China; 3 Analysis center, Tsinghai University, Beijing, 100084 P. R. China; 4 Department of Food Science and Technology, School of Agriculture and Biology, Shanghai Jiao Tong University, Shanghai, 200240 P.R. China

## Abstract

A developed Fourier transform infrared spectroscopy (FT-IR) was employed to investigate changes of protein conformation, which played significant roles in maintaining stable protein networks of white croaker surimi gel, exploring the relationship between protein conformation and surimi gel networks. Spectra of surimi and gels with different grades (A, AA, FA and SA) were analyzed by tri-step FT-IR method and peak-fitting of deconvolved and baseline corrected amide I bands (1600~1700 cm^−1^). The result showed that *α*-helix was the main conformation of surimi proteins. During surimi gelation, *α*-helix of myosin partially transformed into *β*-sheet, *β*-turn and random coil structures. *β*-sheet and random coil structures were the main protein conformations maintaining the structure of surimi gel, of which *β*-sheet made the main contribution to gel strength. Scanning electron microscopy (SEM) result revealed that surimi gels had a fibrous and homogeneous network structure. Moreover, ordered interconnections between three-dimensional proteins networks of gels were inclined to emerge in higher grade surimi, in agreement with the gel strength results. It was demonstrated that the tri-step FT-IR spectroscopy combined with peak-fitting could be applicable for exploration of surimi protein conformation changes during gelation to deepen understanding of its effect on gel quality.

## Introduction

Surimi is a refined fish protein product containing concentrated myofibrillar proteins obtained by deboning, mincing and washing process from fish fleshes for removal of sarcoplasmic proteins, bloods, lipids and enzymes prior to dehydration and blending with cryoprotectants^1^. As an intermediate fish product, it is gaining more prominence because of its high-protein, low-fat and ready-to-cook characteristics^2^. Surimi needs further heating to manufacture various fish gel products sold in markets such as fish tofu, fish balls, imitated crab sticks and kamaboko. Gelation is firstly accomplished under the process of grinding and blending with salt to increase solubility or extractability of myofibrillar proteins, then the resultant paste forms an incompact protein network structure while being set at low temperature (40 °C), finally, elasticity and chewiness are gained after being cooked at higher temperature (90 °C)^3,4^. Seafood analogous products can be made using surimi, reproducing attributes of natural equivalents. For these attributes, gel-forming ability is a crucial functional property determining the unique quality such as sensory and texture^5^. Because of the high importance, improving gel property attracts increasing interests concentrated on influences of fish species, freshness, various additives and heating methods in gel characteristics^6^. So it is useful to have an in-depth understanding of the mechanism that governs surimi gelation, benefiting for expanding the potential fish species and improving gel quality. However, mechanism of gelation is still not reported clearly. It has been recognized that myofibrillar proteins (myosin and actin) become solubilized after adding salt and actin, then the network is formed in heating procedure^7^. For detailed explanation of gelation process, sufficient intermolecular bonds, i.e., hydrogen bonds, disulfide bonds, ionic bond and hydrophobic interactions were investigated^8^. Viscoelasticity changes were studied through storage modulus to explain protein aggregation in a dynamic rheological way^9^. Protein structure in surimi and gels was also explored by Raman spectroscopic technique^3^. In most reported literatures, mechanism of gelation was deduced by using isolated preparation of proteins, dissolving them to obtain suspensions. However, some uncertainty existed in preparation of the model system. For example, helical content of myofibrillar preparation may decrease during handling and storage, and this would cause unreliability in results.

Vibrational spectroscopy is a suitable and rapid method to study the molecular changes in proteins during surimi gelation without complicated preparation. Fourier transform infrared spectroscopy (FT-IR) is a vibrational spectroscopic technique which can monitor changes in molecular structures. A tri-step infrared spectroscopy method including original infrared spectroscopy (IR), second derivative spectra and two-dimensional correlation infrared spectroscopy (2DCOS-IR) could increase the resolution of spectra analysis of complex systems. In particularly, the 2DCOS-IR is capable of revealing molecular interactions among various functional groups and macromolecules^10^. Peak-fitting has been applied as an experimental method for estimating secondary structures of polypeptides and proteins, providing valuable information for some ideal formulations and process parameters^11–15^. Infrared spectra result from the absorption of infrared light energy when the frequencies between light and vibrating chemical bonds (such as stretching and bending motions) are identical. Accordingly, chemical bonds or functional groups are manifested by profiles of spectrum peaks locating at various wavenumbers. Generally, amide bands in infrared spectra are primarily assigned to varied secondary structural compositions of proteins, e.g., amide bands I (80% C=O stretch, near 1650 cm^−1^), II (60% N-H bend and 40% C-N stretch, near 1550 cm^−1^), and III (40% C-N stretch, 30% N-H bend, near 1300 cm^−1^)^15,16^.

The objective of this work was to investigate the structural changes of proteins during gelation of different grades white croaker surimi through tri-step FT-IR and peak-fitting methods, as well as the observation of their microstructures and texture properties, in order to obtain integral chemical and visual insights into the surimi gelation process involving protein denaturation and the relationship between surimi grades and their gel quality.

## Results and Discussion

### Chemical analysis of surimi

**Figure 1:**
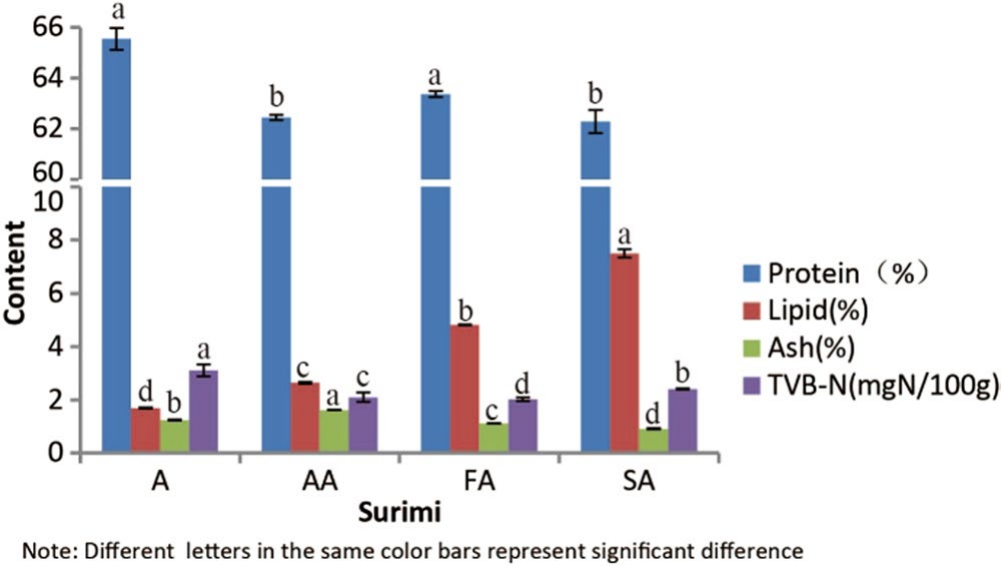
Chemical composition of four grades of surimi.

Chemical compositions of frozen surimi have been analyzed and revealed in Fig. 1. The contents of lipids, proteins, TVB-N (Total volatile basic nitrogen) and ash in different grades of white croaker surimi have significant differences. The lipid content increases from A to SA, corresponding to surimi quality grades, proteins (ranging from 62.28 ± 0.46% to 65.54 ± 0.43% in dry weight) are the major composition for all surimi and A surimi has the highest protein. SA surimi has the lowest ash but FA surimi has the lowest TVB-N. According to national frozen standard of frozen surimi (SCT 3702-2014, China), the classification of surimi quality is based on gel strength, number of impurity and water content, so it is uncertain to conclude that these chemical compositions (lipids, proteins,ash and TVB-N) have any relationship with surimi grades, but it may be useful to investigate the gel-forming process of surimi.

### IR spectra of four grades of surimi and gels

As IR spectrum is molecular vibrational spectrum, the frequencies or wavenumbers of bands is closely determined by the types of chemical bonds and their modes of vibration. Condition changes, such as temperature or aqueous solvent replacement by deutoxide, will lead to conformation alteration of proteins, e.g., peptide-bond angles and hydrogen-bonding, resulting in a rise or decrease to precise wavenumbers of amide bands. Thus, IR can be used to study the protein structures in the base of wavenumber shift and intensity calculation of peaks^16^. Thermal gelation is essentially a result of protein denaturation, i.e., intermolecular and intramolecular covalent and non-covalent interactions^4^. Surimi mainly contains proteins in dry weight in which myosin is the most pivotal component to form the cross-linking protein network. Generally, the absorption bands in amide I (~1655 cm^−1^) and II (~1545 cm^−1^) are prevalently applied despite other bands could provide valuable information as well. Detailed assignments of characteristic absorption peaks in IR spectra of surimi are summarized in Supplementary Table 1.

**Figure 2:**
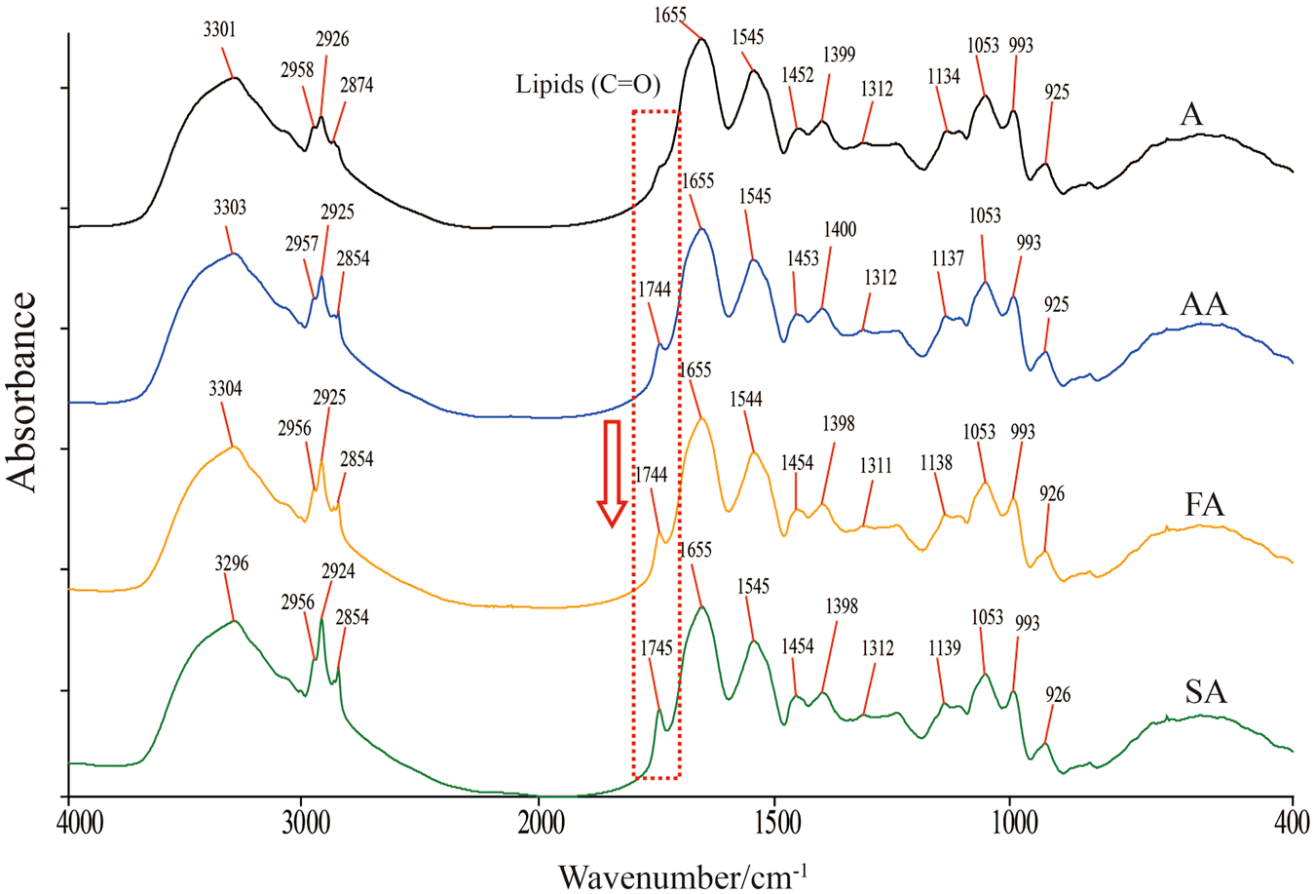
IR spectra of A, AA, FA and SA surimi.

Figure 2 is the spectra of four kinds of surimi, it is expected they are very similar because of the same fish species. However, the intensity of characteristic peak at 1744 cm^−1^ gradually increased from A to SA, suggesting that lipid content in all samples (A, AA, SA, FA) should increase accordingly, and this is verified by former chemical analysis in Fig. 1. Surimi gel spectra (Supplementary Figure 1) also have very high similarity with raw surimi. The bands of 1053 cm^−1^, 993 cm^−1^ and 925 cm^−1^ belong to C-O stretching, mainly contributed by sucrose added during processing. Peaks of lipid and sucrose have the same peak shape and peak area intensity, indicating that the contents of them almost change little before and after gelation, so it can be inferred preliminarily that lipid and sucrose have little effect in surimi gel-forming. However, the absorption bands of amides I and II have been significantly broadened (20%) after gelation, indicating a protein structural change during gelation process, for example, reduction of hydrogen bond^17^. Because the temperature is gradually increased, hydrogen bonds, ionic bonds and other weak bonds in proteins are probably broken.

### Second derivative IR spectra of four grades of surimi and gels

**Figure 3:**
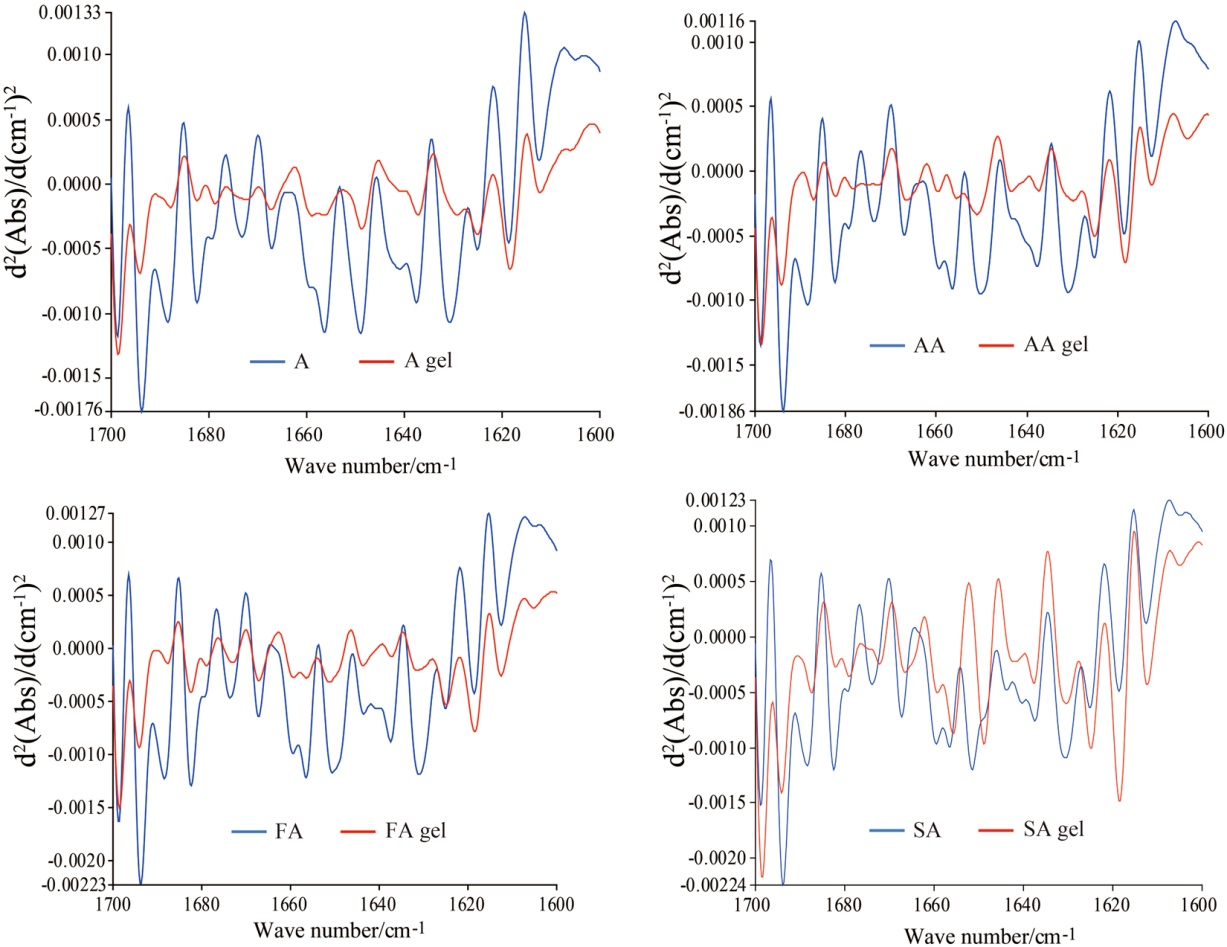
Second derivative spectra of surimi and gels in the region of 1700~1600 cm^−1^.

Second derivative IR spectroscopy is generally used to separate some overlapped absorption peaks and shoulder peaks that are not distinguished in original spectra^10^. Supplementary Figures 2 and 3 show the second derivative spectra of surimi and gels with different grades. Peaks at 1656 cm^−1^, 1630 cm^−1^, 1563 cm^−1^ and 1514 cm^−1^ are weaker in surimi gels than that in surimi, while surimi gels have stronger absorption peaks at 1501 cm^−1^. Figure 3 is the comparison of second derivative spectra of surimi and surimi gels in the region of 1700~1600 cm^−1^. Significant differences could be observed and specific changes could be obtained in further study.

### 2DCOS-IR spectra of four grades of surimi and gels

Two-dimensional correlation infrared spectroscopy (2DCOS-IR) was employed in the range of 1680–1600 cm^−1^ in order to identify differences among the surimi and gels more convincingly. 2DCOS-IR analysis considerably enhances resolution of spectra by providing further information on molecular structures and interactions of functional groups within and between molecules, showing the influences of perturbation on vibration of molecular relative groups in research system components^18^. The peaks (auto-peaks and cross-peaks) in 2DCOS-IR represent the coincidence of spectral intensity variations at corresponding variables along the perturbation and can be used to authenticate differences between samples^19^. Red or green area refers to positive correlation, indicating a group of absorption bands change simultaneously (either stronger or weaker), while blue area is just the reverse^20^.

**Figure 4:**
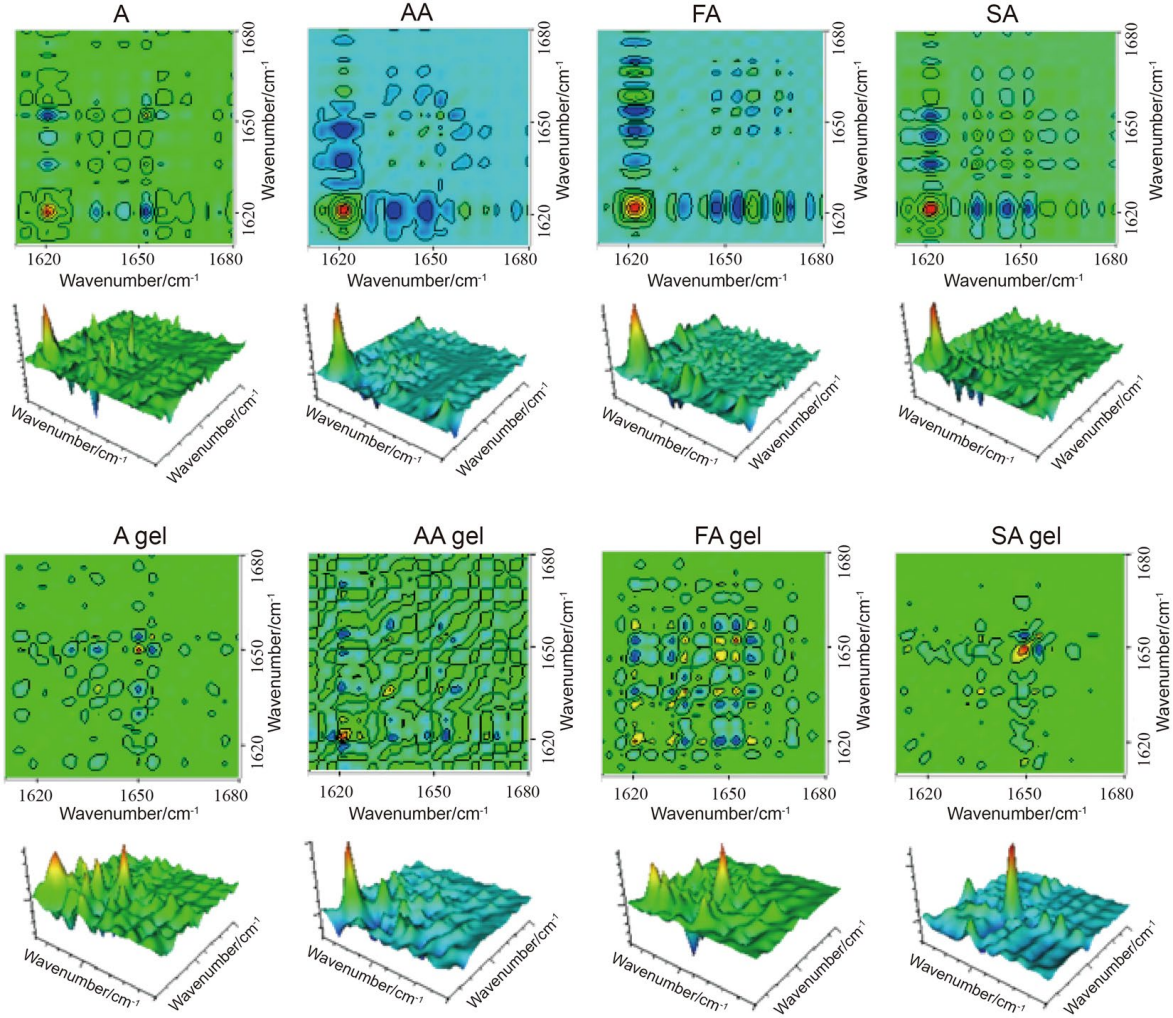
2DCOS-IR synchronous spectra of surimi and gels in the region of 1600~1680 cm^−1^.

The differences among the surimi and gels could be described further through the synchronous 2DCOS-IR spectra. Figure 4 is the synchronous 2DCOS-IR spectra of four grades of surimi and gels in the range of 1680~1600 cm^−1^. It is visualized that peaks vary differently between surimi and gels, and the results could be observed clearly in Table 1, in which peak positions and correlation of auto-peaks are summarized. A surimi has one strong auto-peak at 1621 cm^−1^ while A gel has two strong auto-peaks at 1621 cm^−1^ and 1649 cm^−1^. AA has one strong auto-peak at 1621 cm^−1^ while AA gel has one strong auto-peak at 1623 cm^−1^. FA has one strong auto-peak at 1622 cm^−1^ while FA gel has three strong auto-peaks at 1622 cm^−1^, 1636 cm^−1^ and 1649 cm^−1^. SA has one strong auto-peak at 1621 cm^−1^ while SA gel has one strong auto-peak at 1649 cm^−1^. From comparison of surimi and gel auto-peaks, surimi and gels have different profiles of proteins that response differently to heat perturbation. Gelation is a result of protein denaturation, the process entails the association of long myofibrillar protein chains which produces a continuous three-dimensional protein network^21^. We discover surimi gelation contains some protein conformation changes, and we need a quantitative measure to show these changes in the protein amide bands.

**Table 1:** Auto-peaks in 2DCOS-IR synchronous spectra of surimi and gels.

Samples		Autopeaks/cm^−1^ (Threshold 50% of relative intensity)									
**Surimi**	**A**	**1621**	1630	1636	1644	1652	1656	1685			
	**AA**	**1621**	1630	1639	1647	1652	1657	1667	1686	1694	
	**FA**	**1622**	1634	1638	1643	1647	1653	1658	1666	1670	1676
	**SA**	**1621**	1632	1636	1640	1645	1653	1659	1667		
**Gels**	**A**	**1621**	1628	1638	**1649**	1657	1682				
	**AA**	**1623**	1637	1642	1659	1674	1685				
	**FA**	**1622**	1630	**1636**	1642	**1649**	1659				
	**SA**	1624	1629	1634	1638	**1649**	1656	1682			

Notes: Peaks in bold are strong auto-peaks.

### Amide I band components for surimi and gels

The amide I band, between 1600 and 1700 cm^−1^, is very useful for infrared spectroscopic analysis of secondary structures of proteins, they are usually reflected by the components as follows: 1610~1640 cm^−1^ and 1670~1690 cm^−1^ for *β*-sheet; 1640~1650 cm^−1^ for random coil; 1650~1658 cm^−1^ for *α*-helix; 1660~1700 cm^−1^ for *β*-turn^22,23^. Quantitative peak-fitting analysis of amide I band, as applied in this investigation, has been proved useful in studying the nature and the extent of protein conformation changes^14,16^. Peak-fitting is conducted based on the assumption of Gaussian peak^11^ and that extinction coefficients for all structural elements are the same, so the amide band intensities are proportional to the fraction of each secondary structure^16^. The peak areas are calculated to identify the secondary structure contents in different samples, the component peaks and their locations are shown in Supplementary Table 2 and Supplementary Figure 4, the result of secondary structure contents is listed in Table 2.

**Table 2:** Contents of the secondary structure in surimi and gels with different grades (n = 3).

Samples		Percent (%)			
		α-Helix	β-Sheet	Turn	Random Coil
**Surimi**	**A**	44.79 ± 3.21	32.98 ± 2.15	7.96 ± 1.22	14.27 ± 0.98
	**AA**	43.94 ± 4.69	33.94 ± 1.26	7.92 ± 0.95	14.20 ± 1.25
	**FA**	44.57 ± 3.52	33.39 ± 2.46	7.75 ± 1.36	14.29 ± 2.01
	**SA**	44.18 ± 2.30	33.74 ± 1.23	7.747 ± 1.25	14.347 ± 2.37
**Gels**	**A**	14.617 ± 2.86	43.687 ± 4.45	13.287 ± 2.65	28.437 ± 3.75
	**AA**	13.657 ± 1.26	45.107 ± 2.38	12.097 ± 1.63	29.167 ± 2.58
	**FA**	13.617 ± 0.94	46.187 ± 3.46	12.207 ± 1.45	28.017 ± 1.96
	**SA**	14.497 ± 1.65	44.907 ± 2.72	12.707 ± 2.29	27.91 ± 1.72

Note: Values in the table denote the mean ± standard error.

One major observation is that *α*-helix is the main conformation of surimi proteins, and the content of *α*-helix is reduced and contents of *β*-sheet, *β*-turn and random coil structures are increased, indicating that *α*-helix of myosin partially changes into *β*-sheet, *β*-turn and random coil during surimi gelation. This transition involving rearrangement of protein hydrogen bonding is in agreement with the findings in similar experimental conditions previously reported^24^. The percentage decrease of *α*-helix is regarded as an indicator of partial denaturation of proteins^25^. The increased content of *β*-sheet could be a result of gelation process occurring at low temperature. Some investigations suggested a relationship between the liberation of water and formation of *β-*sheet during heating to higher temperature, because as temperature increased in subsequent cooking, portion of bound water from peptide carbonyl groups was released^21,26^.

### Texture analysis

As Table 3 shows, gel strength is increased from A to SA, higher grade surimi has better surimi gels, and this may be attributed to the difference in profile of proteins in surimi with varied quality grades. In combination with Table 2, it can be inferred that *β*-sheet and random coil structures are the main protein conformations maintaining the framework of surimi gel, and *β*-sheet makes the main contribution to gel strength. The elasticity of surimi gel is mainly associated with the polymerization of myofibrillar proteins and this gel-forming ability has been proved to be a result of aggregation of myosin heavy chains (MHCs) due to the action of endogenous transglutaminase (TGase)^27,28^. It is reported that *α*-helix in myosin molecules are induced to unfold the coiled-coil structure, especially their rod part, to facilitate the formation of strong thermal gels during heating step^28^.

**Table 3:** Texture properties of surimi gels with different grades (n = 3).

Material	Gel Strength (g·cm)
A gel	492.52 ± 64.65^a^
AA gel	639.35 ± 61.95^b^
FA gel	865.60 ± 41.08^c^
SA gel	1099.89 ± 40.39^d^

Note: Values in the table denote the mean ± standard error. Different superscript letters indicate significant differences (P  < 0.05).

### Microstructure analysis of four grades of surimi and gels

**Figure 5:**
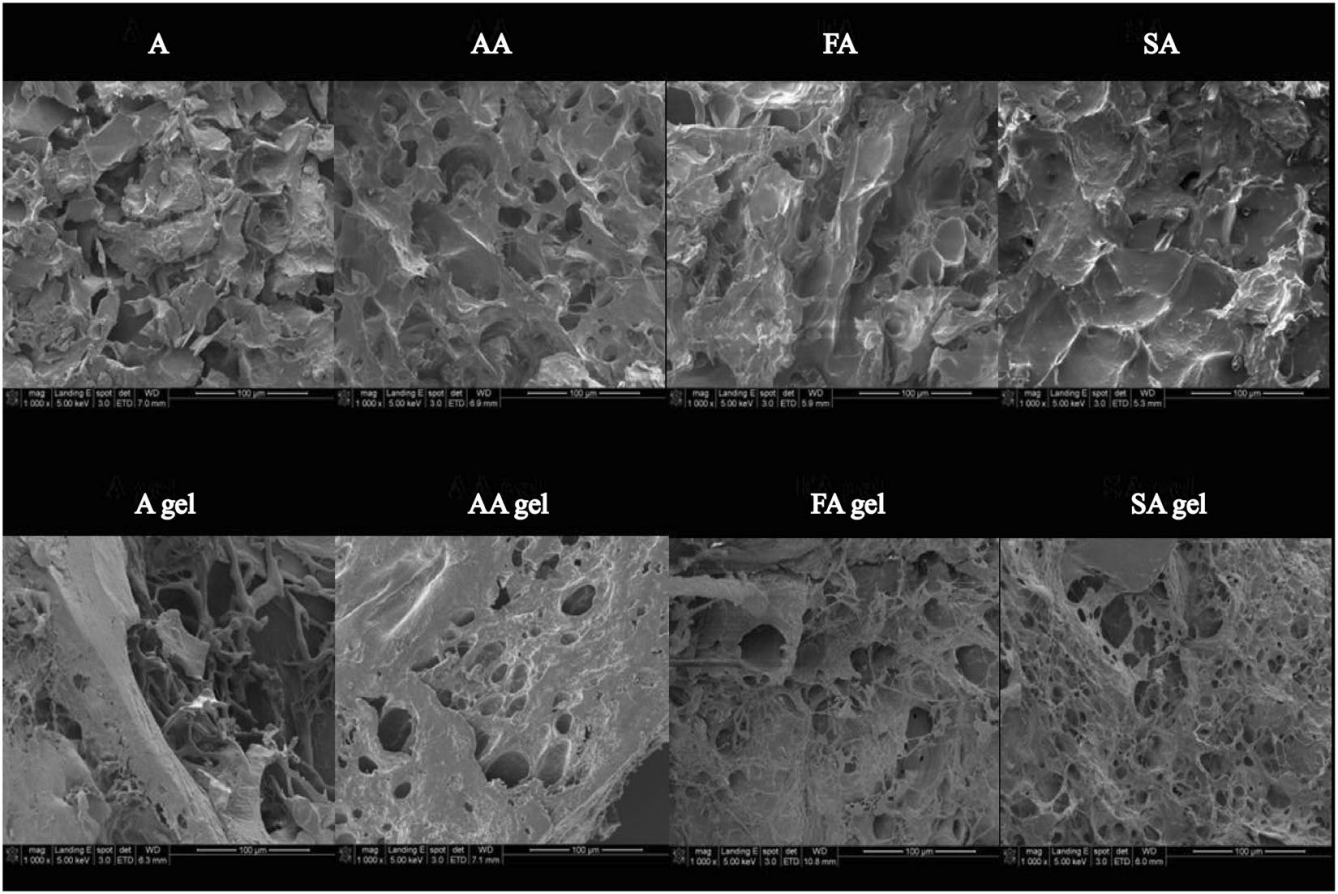
Scanning electron micrographs (1000 × magnifications, bar 100 µm) of surimi and gels with different grades.

To verify the connection between protein conformational changes and gel strength, the microstructures of surimi and gels were observed with a SEM. The photographs of surimi display a smooth protein matrix with fewer but bigger cavities than gels, and the gels show that all of them have network structures, which allows the surimi gels to maintain certain elastic characteristic (Fig. 5). In addition, a more compact and denser gel network with a thin band can be observed in surimi gels of grades AA, FA and SA when compared with that of grade A, mainly for the reason that these thermal gels hold a large volume of water within the myosin molecules network. Therefore, it can be hypothesized that interconnections between the three-dimensional protein networks of surimi gels are inclined to emerge in higher grade gels and thus contribute to their texture quality such as gel strength. The results are in agreement with the results shown in Table 3 and help to explain the increased gel strength from A to SA surimi gels in a microscopic level.

## Conclusion

During gelation process, hydrogen bond, ionic bonds and other weak bonds in protein molecules are broken, resulting in the destruction of protein structures. The changes of protein secondary structures are the decrease of *α*-helix and the increase of *β*-sheet, *β*-turn and random coil structures. Gel-forming ability is related to the conversion that *α*-helix transforms into other types of secondary structures, such as *β*-sheet and random coil, and *β*-sheet contributes more to increase gel strength. High grade surimi forms a more compact and denser protein network structures which accounts for its high gel strength. It has been demonstrated that the tri-step FT-IR spectroscopy combined with peak-fitting could be a scientific and rapid tool for in-depth investigation of surimi protein conformation changes during gelation.

## Methods

### Apparatus and samples

The spectrometer was Thermo Scientific Nicolet iS5 equipped with a DTGS detector and the spectra were recorded with 16 scans in the range of 4000~400 cm^−1^ at a resolution of 4 cm^−1^. The interferences of H_2_O and CO_2_ were subtracted when spectrum acquisition. The samples were set on the heated plate of the multiple reflection Horizontal ATR (Attenuated Total Reflectance) units which contained two cartridge heaters to heat the crystal plate and ensure even heating. The temperature of the block was monitored and controlled by a Resistive Thermal Detector. SMS TA XTPlus (Stable Micro Systems, UK) was employed for texture analysis.

Muffle furnace SXL-1002 (Shanghai Jing Hong Experimental Equipment Co., China); Kjeltec 8400 Analyzer Unit (Foss, Sweden); Blast Oven DHG-9140A (Shanghai HuiTai Instrument Manufacturing Co., China); Freeze Dryer BTP-3XLOVX (Virtis, American); Soxtec2050 (FOSS, Denmark).

Samples of four kinds of frozen white croaker (*Argyrosomus argentatus*) surimi, differentiating in quality grades (A, AA, FA and SA, sequence follows increasing quality), were brought from Zhejiang Zhoushan Food Company (Zhoushan, Zhejiang Province, China).The quality grades were determined by gel strength, number of impurity and water content set in the national frozen surimi standard (SCT 3702-2014, China).

### Chemical composition analysis

Protein content was determined by constant Kjeldahl method (AOAC 981.10, 2007) measuring nitrogen (N × 6.25). Fat content was extracted by Soxhlet apparatus (AOAC 960.39, 2007) using ether as solvent and estimated as free fat. Ash content of the sample was determined by method named Muffle furnace ashing (AOAC, 938.08, 2007). TVB-N content was estimated by automatic Kjeldahl analyzer with 5.0 g of each sample and 0.5 g Magnesia were placed in digestive tract.

### Surimi gel preparation

Frozen white croaker surimi was thawed in 4 °C and cut into small chunks, those chunks were ground in a universal food processor at low speed for 3 min. Then salt (3% w/w) was added for three times in 8 min and meanwhile the surimi paste was chopped at the same speed under a temperature maintaining below 5 °C. The salt-ground surimi was stuffed into polyvinylidene chloride casing and the suwari treatment was accomplished by two steps, i.e., firstly set in a water bath at 40 °C for 60 min and then cooked at 90 °C for 30 min. Afterwards these set-cooked surimi gels were chilled immediately with flowing ice water. Finally, all samples were dispensed into a vacuum bag and stored in a refrigerator at 4 °C before analysis^29^.

### Measurement of gel properties

The casings of surimi gels were stripped, samples were cut into cylinders of a constant dimension (32 mm diameter, 25 mm height), and then positioned diametrically under the 5 mm Spherical Probe (P/5 S) to commence the penetration test. Once the trigger force of 10 g was attained, the probe proceeded to penetrate the sample to a distance of 15 mm. The force puncturing into surimi gel (breaking force) and the distance at which the ball probe punctured into it (breaking distance) were both important parameters. The texture property was measured according to three parameters, maximum force (breaking force), distance to rupture and “gel strength”^28^. “Gel strength” was the peak force in grams, multiplied by breaking force and breaking distance.

### Infrared spectroscopic analysis

Frozen white croaker surimi was removed to 4 °C refrigerator and thawed overnight, then the surimi and surimi gels were freeze-dried for 24 h for pulverizing into fine powders, finally they were blended with 1~2 mg KBr (Potassium bromide) and pressed into a tablet. All FT-IR spectra were collected at a condition of low air humidity (below 40%) and room temperature by Thermo FT-IR spectrometer. The raw FT-IR data was processed with Omnic spectrum software (Version 9.2.106) and PerkinElmer spectrum software (Version 6.3.5), second derivative IR spectra were obtained after 13-point smoothing pretreatment on corresponding original IR spectra. For 2DCOS-IR acquisition, ATR accessory connecting with a temperature controller was used to collect dynamic original spectra of different temperatures at an interval of 5 °C in the range from 30 to 70 °C. 2DCOS-IR spectra were achieved by analyzing the series of original temperature-dependent spectra in 2DCOS-IR correlation analysis software (Nicolet iN10 Spectra Corr). For analyzing secondary structures of proteins, recorded FT-IR spectra of surimi and gels (A, AA, FA and SA) were analyzed by peak-fitting software (Version 4.12) of the deconvolved and baseline corrected amide I bands (1600~1700 cm^−1^).

### Microstructure analysis

Microstructure of the frozen surimi and gels was obtained by scanning electron microscopy (SEM). Samples were cut into slices with a thickness of 2–3 mm, soaked with 3% glutaraldehyde solution and then washed in distilled water for 1 hour prior to being dehydrated with ethanol. Dried samples were placed on a bronze stub and sputter-coated with gold for observation by a scanning electron microscope (Nova NanoSEM 230, FEI, USA) at an acceleration voltage of 5 kV^30^.

### Data availability

The dataset generated or analyzed during the current study are available from corresponding author upon reasonable request.

## Electronic supplementary material

Supplementary information
