# Supplementary material for: Analysis of protein structure changes and quality regulation of surimi during gelation based on infrared spectroscopy and microscopic imaging
Source: Sci Rep. 2018 Apr 3;8:5566. doi: 10.1038/s41598-018-23645-3 (PMC5882903; doi:10.1038/s41598-018-23645-3)
Supplement: Supplementary file 1 — Supplementary information [file 41598_2018_23645_MOESM1_ESM.docx]

**Analysis of protein structure changes and quality regulation of surimi during gelation based on infrared spectroscopy and microscopic imaging**

Wei Wei^1#^, Wei Hu^1#^, Xian-Yi Zhang^1^, Feng-Ping Zhang^2^, Su-Qin Sun^3^, Yuan Liu^4^, Chang-Hua Xu^1,3^^[[1]](#footnote-1)^*

1. College of Food Science & Technology, Shanghai Ocean University, Shanghai 201306, P.R. China
2. Tongwei Co., Ltd., Chengdu, Sichuan, 610041, P.R. China
3. Analysis center, Tsinghai University, Beijing 100084, P.R. China
4. Department of Food Science and Technology, School of Agriculture and Biology, Shanghai Jiao Tong University, Shanghai, 200240, P.R. China

**Supplementary figures**


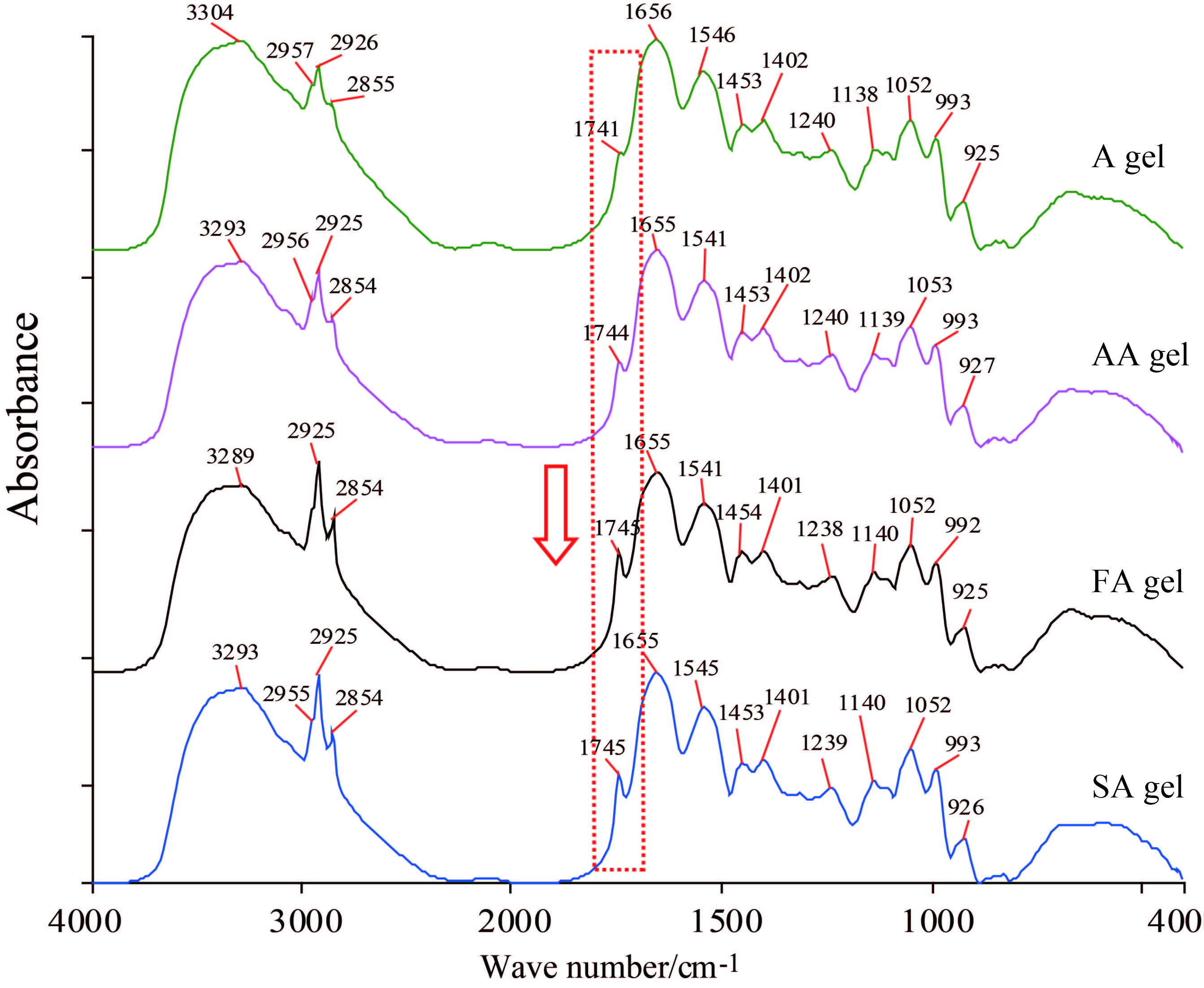


**Supplementary Figure 1** IR spectra of A, AA, FA and SA surimi gels


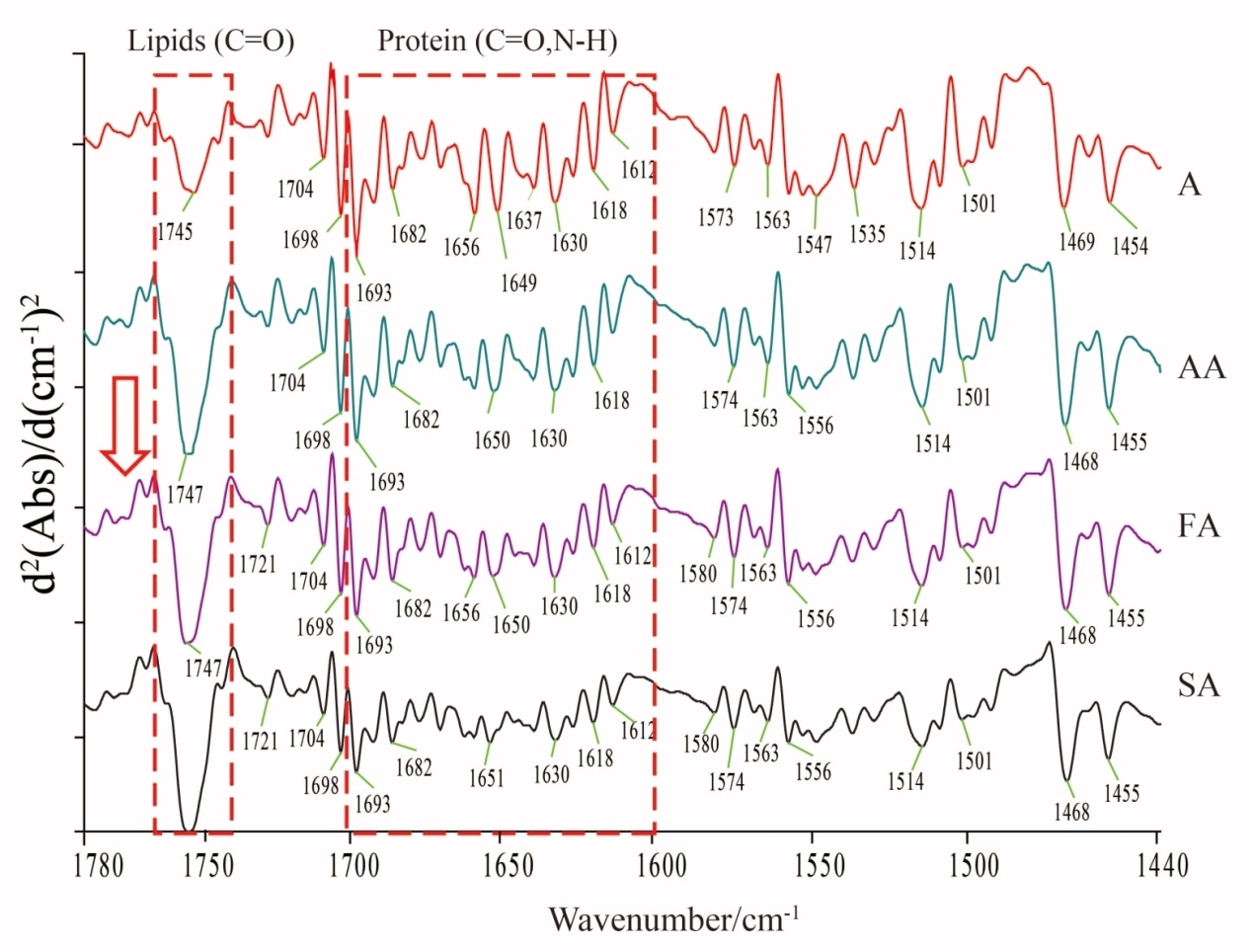


**Supplementary Figure 2** Second derivative spectra of A, AA, FA and SA surimi


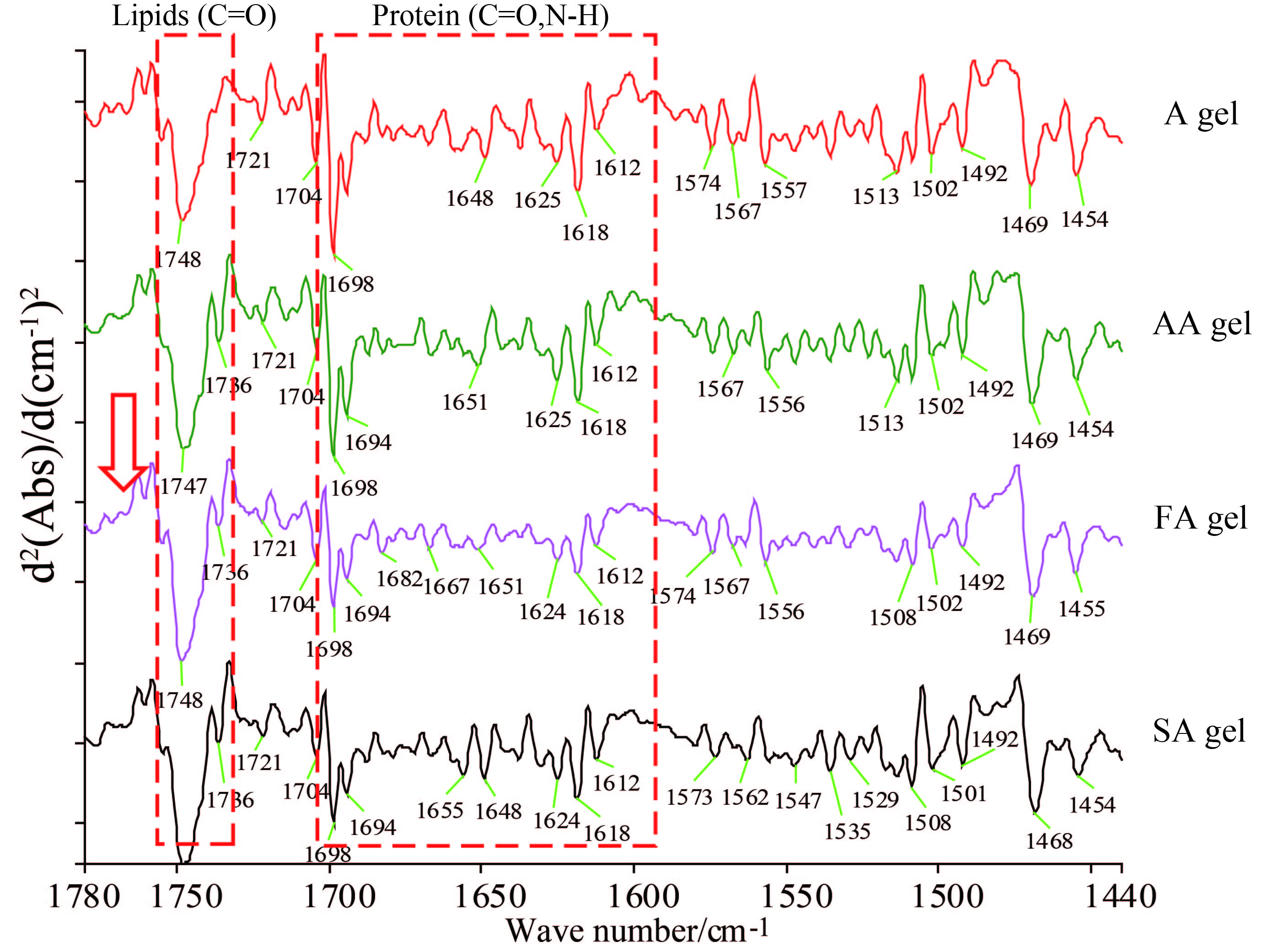


**Supplementary Figure 3** Second derivative spectra of A, AA, FA and SA surimi gels


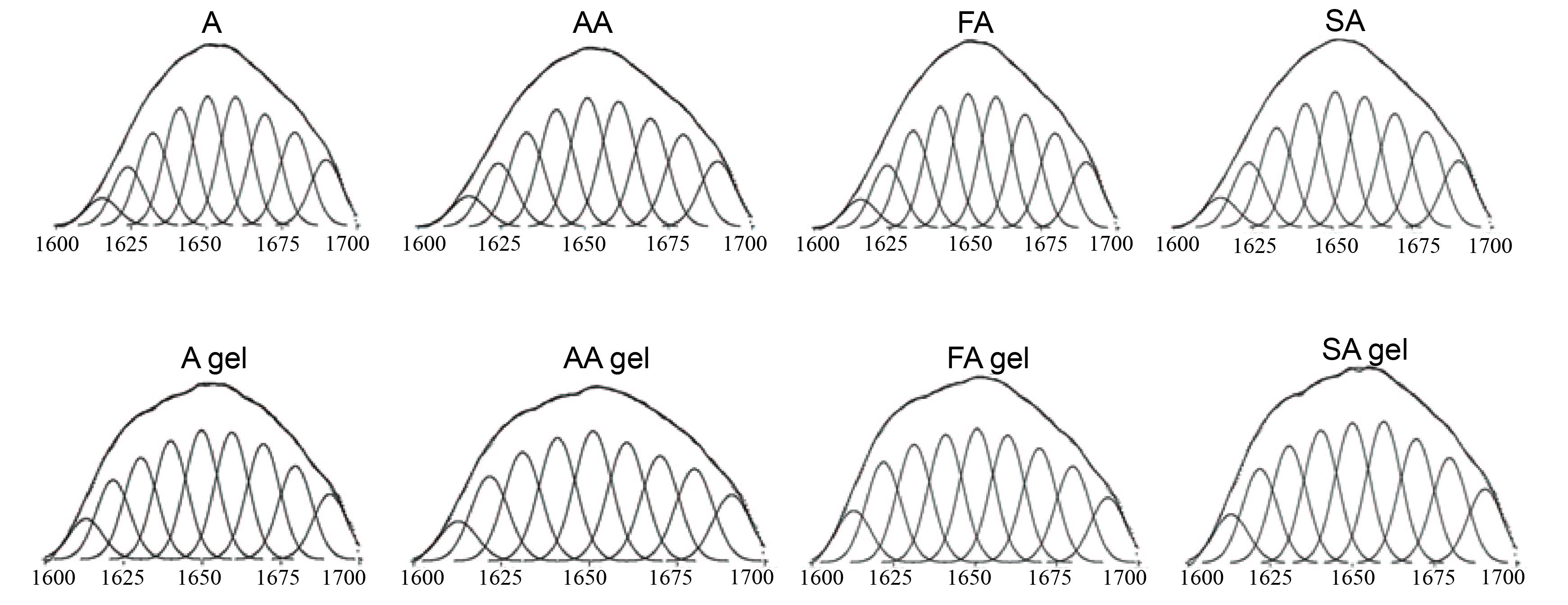


**Supplementary Figure 4** Peak-fitting of amide I band of surimi and surimi gels spectra in the region of 1700~1600 cm^-1^

**Supplementary tables**

**Supplementary Table 1** The preliminary assignment of main characteristic absorption peaks of FT-IR spectra of surimi and gels

| Peak position (cm^-1^) | Vibration mode | Main attribution |
| --- | --- | --- |
| 2956, 2925, 2872, 2854 | *v_as_*(C-H) | CH_2_, CH_3_ |
| 1744 | *v_s_*(C=O) | carbonyl group |
| 1655 | *v*(C=O) | protein |
| 1545 | *v*(C-N) | protein |
| 1454 | *v_as_*(C-H), *v_s_*(C-H) | CH_3_ |
| 1053, 993, 925 | ν(C-O) | sucrose |
| Note: *v*, stretching mode; *s*, symmetric; *as*, asymmetric | | |

**Supplementary Table 2** Location of amide I components for surimi and gels

| **Samples** | | **Component peak locations (cm^-1^)** | | | | | | | | |
| --- | --- | --- | --- | --- | --- | --- | --- | --- | --- | --- |
|  |  | 1 | 2 | 3 | 4 | 5 | 6 | 7 | 8 | 9 |
| **Surimi** | **A** | 1615 | 1623 | 1632 | 1641 | 1650 | 1657 | 1669 | 1679 | 1689 |
|  | **AA** | 1615 | 1624 | 1632 | 1641 | 1650 | 1655 | 1669 | 1679 | 1689 |
|  | **FA** | 1615 | 1624 | 1632 | 1641 | 1650 | 1656 | 1669 | 1679 | 1689 |
|  | **SA** | 1614 | 1623 | 1632 | 1641 | 1650 | 1653 | 1669 | 1679 | 1689 |
| **Gels** | **A** | 1613 | 1621 | 1630 | 1640 | 1649 | 1659 | 1669 | 1679 | 1690 |
|  | **AA** | 1612 | 1621 | 1631 | 1641 | 1651 | 1660 | 1670 | 1680 | 1690 |
|  | **FA** | 1612 | 1622 | 1631 | 1641 | 1650 | 1660 | 1669 | 1679 | 1690 |
|  | **SA** | 1613 | 1621 | 1630 | 1640 | 1650 | 1659 | 1669 | 1679 | 1690 |
| **Note:** Fit quality (r^2^) between original and fitted spectra ≥0.9998. | | | | | | | | | | |

1. # These authors contributed equally to this work.

   * Corresponding author. E-mail address: [chxu@shou.edu.cn](mailto:chxu@shou.edu.cn) [↑](#footnote-ref-1)
